# Hard Contact Lens Wear and the Risk of Acquired Blepharoptosis: A Case-Control Study

**Published:** 2013-06-19

**Authors:** Takeshi Kitazawa

**Affiliations:** Department of Plastic and Reconstructive Surgery, Matsunami General Hospital, Gifu, Japan

## Abstract

**Objectives:** Since there are increasing numbers of patients with blepharoptosis who have a history of wearing contact lenses, we attempted to estimate the risk of developing ptosis from wearing hard contact lenses. **Methods:** In an age-matched case-control study that was performed in a hospital in Japan, we compared the rate of hard contact lens users in ptosis cases with that in a control group and then estimated the odds ratio. **Results:** The history of wearing hard contact lenses was significantly higher in patients (90.2%) versus controls (31.6%). Hard contact lens wearers had a 20 times increased risk of ptosis (odds ratio: 19.9; 95% confidence interval: 6.32-62.9) compared with the nonwearing subjects. **Conclusions:** This study indicated that there was a significant association between hard contact lenses and blepharoptosis. Because of both the prevalence of use and the aging of the population, contact lens–induced blepharoptosis is no longer just a problem for young and middle-aged people with myopia but also for the elderly population.

Prolonged contact lens wear is well known to potentially cause acquired ptosis.^1^^-^5 Although a few cases have been reported for soft contact lenses,^6^^,^^7^ this is chiefly known to be caused by hard contact lenses. Contact lens–induced ptosis can be diagnosed for contact lens wearers when they present with acquired ptosis without any history of ophthalmic surgery, disease, muscular and neural disorders, or trauma. Moreover, involutional ptosis can be ruled out in very young subjects. The pathogenesis of contact lens–induced ptosis is aponeurogenic and is similar to the involutional changes that are associated with the attenuation or disinsertion of the levator aponeurosis from its distal insertion in the eyelid.

During the aging process, a gradual development of ptosis inevitably occurs in everyone. Thus, how strongly the use of contact lenses affects the progress of ptosis is a great concern for both myopia patients and clinicians. Therefore, the overall goal of this study was to determine the risk of developing ptosis due to wearing hard contact lens. To accomplish this aim, we examined both the number of wearers and the duration of use of hard contact lenses in patients with ptosis and then compared these findings with sex- and age-matched controls.

## METHODS

### Patients and controls

**Figure 1:**
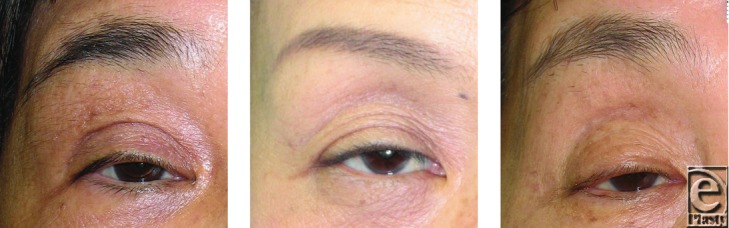
Representative cases of patients with margin reflex distance of 1.5 mm or less who had a history of hard contact lens wearing for more than 30 years.

**Figure 2:**
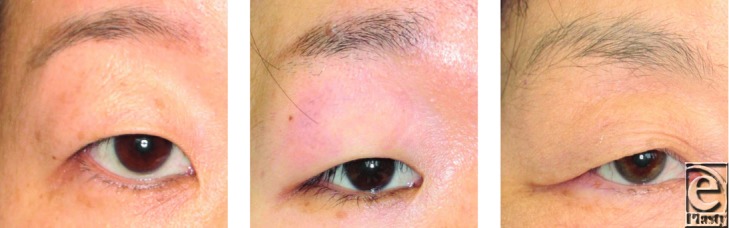
Representative cases of suspected ptosis excluded from patients. Although the patients had a margin reflex distance that was less than 1.5 mm because of sagging anterior lamella, there was also a fully retraced posterior lamella of the eyelid.

**Figure 3:**
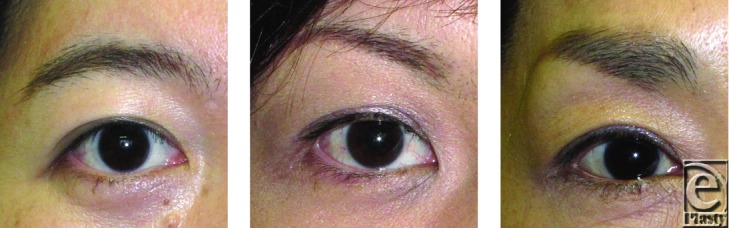
Representatives of controls with MRD of 3.0 mm or more.

This study included all patients aged between 30 and 60 years who were seen with aponeurotic ptosis in the Department of Plastic and Reconstructive Surgery at Matsunami General Hospital between April 2009 and March 2012. *Ptosis* was defined as a margin reflex distance (MRD) of both eyes that was less than or equal to 1.5 mm (Fig 1). Eyes with suspected ptosis in which the MRD narrowed to less than 1.5 mm because of sagging upper lid skin but for which there was an acceptable elevation of the gray line (true lid margin) were not included in these analyses (Fig 2). *Controls* were defined as subjects with an MRD of both eyes that was more than or equal to 3 mm (Fig 3). Control subjects were selected from an age-matched group of female hospital employees. Patients with a history of congenital ptosis, ophthalmic surgery, muscular or neurogenic disorders, and trauma were excluded. In the control group, subjects who had previously undergone eyelid surgery were also excluded from the study. As most of the patients with ptosis were female, we additionally excluded male subjects from this study to simplify the statistical analysis.

After enrolment, all patients and controls were questioned about past contact lens wear, including duration and type of contact lenses worn, and any history of other possible causes for the acquired ptosis. Subjects were photographed together with a scale beside their eyes, using a camera mounted with a flash that ensured there was a catchlight in the center of their pupil when they gazed at the lens. These pictures were subsequently used to bilaterally measure the MRD and the palpebral fissure height. The levator function was determined by measuring the excursion of the eyelid margin as they looked from a downgaze to an upgaze position. During the measurements, the eyebrow was manually fixed against the supraorbital rim to prevent any contribution from the frontal muscle. All participants provided their written informed consent before participating in this study.

Since the patients and controls were distributed normally, a Student unpaired *t* test was used to compare the mean age difference between the groups. The Mann-Whiney *U* test was used to compare the mean MRD, palpebral fissure height, and levator function between the 2 groups because of their nonnormal distribution. A χ^2^ test was used to estimate the odds ratio of the rate of the hard contact lens usage between the 2 groups. Stat View for Windows version J-5.0 (SAS Institute, Cary, NC) was used for all of the statistical analyses.

## RESULTS

Table 1 presents the design of current study, while Table 2 lists the subjects’ demographic data. A total of 51 patients and 38 controls were interviewed and photographed and underwent measurements of their bilateral eyelids. Mean age was 48.1 years with a standard deviation of 7.8 years in the patients, while it was 47.3 years with a standard deviation of 8.4 years in the controls (*P* = .67). A considerably higher rate of experience for the hard contact lens was observed in the patients (90.2%) than in the controls (31.6%). The average period of time of wearing contact lens was longer in the patients (29.6 years) than in the controls (23.2 years). Statistical analysis showed a significant association between the history of hard contact lens wear and acquired ptosis (odds ratio: 19.9; 95% confidence interval: 6.32-62.9; *P* < .001; Table 3).

## DISCUSSION

The current findings indicated that there was a possible relationship between wearing hard contact lens and blepharoptosis. These results showed that women wearing hard contact lens had a 20 times higher risk of ptosis than nonwearers. Previous publications have also demonstrated an association between wearing contact lenses and acquired ptosis. A study by van den Bosch and Lemij^2^ found that the mean MRD for hard contact lens wearers was 0.5 mm smaller than that of the control group. Fonn et al^8^ reported that palpebral aperture size, which is the same as the palpebral fissure height, for the hard contact lens wearers was 0.48 mm smaller than that of the soft contact lens wearers and 0.34 mm smaller than that of nonwearers. Although previous studies have compared contact lens wearers with nonwearers, the current study may be the first to compare ptosis patients with controls.

In our present study, we determined that 90% of the ptosis patients had a history of wearing hard contact lenses. This value is much greater than that of other previous studies, which have reported a range from 7% to 47%.^3^^,^^5^ Although these values may be due to differences in the prevalence or preference of hard contact lenses or perhaps due to regional differences in the myopic population, we speculate that our considerably higher percentage may have been due to different ptosis criteria. The criteria adopted in our study were based on the definition by Small et al,^9^ who defined ptosis as an MRD of 1.5 mm or less. Using an MRD of 1.5 mm was chosen as the cutoff because even Japanese subjects at this level can exhibit a droopy eye look, with many of them presenting a typical Mongoloid eye characterized by a puffy eyelid and narrow palpebral fissure. However, the criteria used by the other studies included an MRD of less than 2.8 or 2.5 mm.^2^^,^^10^ If we had adopted these levels for our current study, this could have led to a large number of Japanese without subjective symptoms being classified as having ptosis. Therefore, we surmise that our restrictive criteria prevented the inclusion of potential ptosis caused by other factors, thereby making it possible to analyze severe ptosis directly caused by the use of hard contact lenses.

Contact lens–induced ptosis is considered to be aponeurogenic and similar to involutional ptosis. In fact, disinsertion, dehiscence, or rarefaction of the levator aponeurosis can be observed intraoperatively, with the ptosis reduced by surgical repair of the affected aponeurosis. At the present time, aponeurotic ptosis that occurs in elderly patients without any other causes is considered to be involutional, while it is diagnosed as contact lens–induced ptosis when it occurs in young and middle-aged patients who have a history of wearing contact lenses. However, since today many older patients also have a history of wearing contact lenses, the question that now needs to be answered is whether the ptosis found in these elderly patients is involutional or contact lens–induced? Thus, as the number of subjects who wear contact lenses in the Japanese population continues to rise, the contribution of contact lens wear to the development of ptosis in middle-aged and elderly patients is becoming a much more important issue. Thus, to further evaluate these ongoing changes, the current study attempted to estimate the risk of developing ptosis in subjects who wear hard contact lens.

Although our study did show that prolonged use of hard contact lenses was a prominent risk factor of ptosis, it should be noted that our study only examined Japanese women. Thus, it is unknown whether similar results will be obtained in men or in other populations around the world. In addition, as the number of soft contact lens wearers continues to increase, studies will also need to include this group in any further examinations of this potential risk.

## Figures and Tables

**Table 1:** Study design: matched case-control

	Ptosis	Control	Total
HCL history (+)	46	12	58
HCL history (−)	5	26	31
Total	51	38	89

Abbreviation: HCL, hard contact lens.

**Table 2:** Subject demographic data*

Variable	Ptosis (*n* = 51, 102 eyelids)	Control (*n* = 38, 76 eyelids)	*P*
Age, y	48.1 ± 7.8	47.3 ± 8.4	.67
MRD, mm	0.5 ± 0.8	3.5 ± 0.6	<.001
PFH, mm	6.3 ± 1.2	9.2 ± 1.2	<.001
LF, mm	11.3 ± 3.2	15.1 ± 1.4	<.001
HCL user, n (%)	46 (90.2)	12 (31.6)	<.001
Duration of use, y	29.6 ± 8.0	23.2 ± 8.3	<.016

Abbreviations: HCL, hard contact lens; LF, levator function; MRD, margin reflex distance; PFH, palpebral fissure height.

*Continuous variables are presented as mean ± standard deviation. Categorical variables are presented as number and percent.

**Table 3:** Odds ratio and 95% confidence intervals of blepharoptosis in relation to hard contact lens wearing

	Odds ratio	95% CI	*P*
HCL wear	19.9	6.32-62.9	<.001

Abbreviations: CI, confidence interval; HCL, hard contact lens.
